# Scene-Aware Adaptive Updating for Visual Tracking via Correlation Filters

**DOI:** 10.3390/s17112626

**Published:** 2017-11-15

**Authors:** Fan Li, Sirou Zhang, Xiaoya Qiao

**Affiliations:** Department of Information and Communication Engineering, School of Electronic and Information Engineering, Xi’an Jiaotong University, Xi’an 710049, China; luminous@stu.xjtu.edu.cn (S.Z.); qxy0212@stu.xjtu.edu.cn (X.Q.)

**Keywords:** visual object tracking, scene-classification, adaptive updating mechanism, occlusion

## Abstract

In recent years, visual object tracking has been widely used in military guidance, human-computer interaction, road traffic, scene monitoring and many other fields. The tracking algorithms based on correlation filters have shown good performance in terms of accuracy and tracking speed. However, their performance is not satisfactory in scenes with scale variation, deformation, and occlusion. In this paper, we propose a scene-aware adaptive updating mechanism for visual tracking via a kernel correlation filter (KCF). First, a low complexity scale estimation method is presented, in which the corresponding weight in five scales is employed to determine the final target scale. Then, the adaptive updating mechanism is presented based on the scene-classification. We classify the video scenes as four categories by video content analysis. According to the target scene, we exploit the adaptive updating mechanism to update the kernel correlation filter to improve the robustness of the tracker, especially in scenes with scale variation, deformation, and occlusion. We evaluate our tracker on the CVPR2013 benchmark. The experimental results obtained with the proposed algorithm are improved by 33.3%, 15%, 6%, 21.9% and 19.8% compared to those of the KCF tracker on the scene with scale variation, partial or long-time large-area occlusion, deformation, fast motion and out-of-view.

## 1. Introduction

In recent years, with the maturation of tracking technology, visual object tracking has been widely used in many fields, such as military guidance, human-computer interaction, road traffic and scene monitoring [1,2]. The goal of visual object tracking is to search for the target in a video sequence. Scholars have also performed a significant amount of research examining tracking algorithms and have produced a number of research results [3,4,5,6,7]. However, in the actual process of tracking, there are many diverse problems, such as scale variation (SV), out-of-plane rotation (OPR), occlusion (OCC), deformation (DEF), motion blur (MB), fast motion (FM), out-of-view (OOV), and background clutter (BC) [8]. These complex and variant target scenes cause the tracking process to be increasingly difficult and affect tracking accuracy. Therefore, how to effectively improve tracking accuracy in these complex scenes is crucial. In addition, actual object tracking always occurs in real time. Thus, ensuring real-time tracking performance is another problem that must be addressed.

To achieve accurate tracking, existing algorithms determine the state of the target in subsequent frames by designing an effective appearance model [3,4,5,6,7,9,10,11,12,13,14,15,16,17,18,19,20,21]. Appearance models are used to describe the target, which can be divided into two categories, generative and discriminative. The tracking algorithms based on the generative model [5,7,9,11,12] usually train a holistic appearance model in a current frame and then the model is matched with the candidate samples in a new frame. These algorithms regard the candidate sample of obtaining the best matching response as the target in the image and then update the appearance model online by using some model updating methods [16]. The majority of generative tracking algorithms obtain the appearance model just in the previous frame, which cannot adapt to the scene with significant appearance change in adjacent frames such as deformation and motion blur. Besides, in the updating process, the algorithms always update the holistic appearance model, which will be likely to fail in the scene with background changes such as occlusion. The IVT algorithm in [5] represents the candidate samples by the mean and feature vectors in the lower dimensional feature space. Then, the model is updated by simple incremental learning, which can adapt to variations in indoor and outdoor environments, such as illumination. However, once the target has partial occlusion or long-time large-area occlusion, the simple incremental learning updating method will incorporate the information of the occluded portion into the model. Thus, when the occlusion disappears, the model will not be able to respond to the target, leading to the tracking drift or failure. The ASLA algorithm in [11] employs template updating strategy that combines incremental subspace learning with sparse representation. This strategy reduces the probability of tracking drift and the influence of occlusion, and can adaptively update the template based on the change to the target appearance.

The tracking algorithms based on discriminative model [10,13,14,18,19,22] usually consider the tracking problem as a classification problem and distinguish the target and background by training and updating classifiers. The classifier is usually trained by the target feature template obtained by extracting the feature from target. The updating of the model is the updating of the target feature template and classifier. Most of the discriminative tracking algorithms exploit both the target and background information, which is useful for tracking accuracy. However, these approaches require a large number of samples to constantly train and update the classifiers online, which will lead to a high computational cost. Therefore, the requirement for real-time performance is difficult to be satisfied.

Since correlation filters is applied to target tracking [15,17,23,24,25,26,27], the speed of discriminative tracking algorithms has improved qualitatively and can reach up to 100 frames per second [15,23,25]. Therefore, the real-time requirement has been guaranteed. In [23], the correlation filter is introduced into the target tracking algorithm for the first time. The algorithm exploits a Minimum Output Sum of Squared Error (MOSSE) filter to track the target. It uses dense samples but has a very fast tracking speed. The CSK tracker proposed in [15], introduces the kernel trick into the correlation filter for the first time. The algorithm improves the linear classifier into the non-linear ridge regression classifier and exploits the highly structured kernel matrix to complete the training sample operations by cyclic shifts. In addition, it exploits the fast Fourier transform, which transforms the correlation operation in the image domain into the multiplication operation in the Fourier domain, greatly reducing the computational complexity. Based on the CSK tracker [15], Henriques et al. [25] proposed the improved tracker KCF, which replaced the gray feature with the HOG feature [28]. This algorithm improves the target description ability and improves the tracking performance. The tracker using a correlation filter has low computational complexity and good real-time performance and has become a focus of tracking research in recent years [20,21,29,30,31,32,33]. In [20], a spatial regularization component is introduced in the learning to penalize correlation filter coefficients depending on their spatial location. The algorithm allows the correlation filters to be learned on a significantly larger set of negative training samples, without corrupting the positive samples, so that can improve the tracking accuracy. In [21], the author equips a basic framework to train a pool of discriminative classifiers jointly in a closed-form fashion. It poses an extra regularization term in ridge regression which interacts with other base models in the ensemble. Through a simple realization of this approach, this paper proposed the co-trained kernelized correlation filters (COKCF) which consist of two KCF trackers to improve the tracking accuracy. In [32], the author proposes a kernel cross-correlator (KCC) that breaks traditional limitations. By introducing the kernel trick, the KCC extends the linear cross-correlation to non-linear space, which is more robust to signal noises and distortions. In [33], the algorithm exploits features extracted from deep convolutional neural networks trained on object recognition datasets and adaptively learns correlation filters on each convolutional layer to encode the target appearance, which improves the tracking accuracy and robustness while sacrifices a large portion of the tracking speed.

However, in the tracking process, the accuracy of the majority of the discriminative tracking algorithms based on correlation filter in complex scenes is not satisfactory because of the deformation of the target and the influence of the target scenes. In the tracking process, the majority of existing algorithms based on correlation filter [15,17,23,24,25,26,27,29] always update the holistic target feature template and the correlation filter coefficient at a fixed learning rate regardless of the target scene. For example, in [25], after the correlation filter obtains the position of the target in the current frame, even if the target is in partial occlusion or long-time large-area occlusion, the KCF algorithm will also update the holistic target feature template, resulting in the information from the occluded portion being updated in the target feature template and learned by the correlation filter. When the occluded target reappears, the filter will not find the matching response, leading to tracking drift or failure. When the target appearance changes drastically, the fixed learning rate will make it difficult for the correlation filter to rapidly learn the information of the target variation and adapt to the drastic changes in the target appearance, resulting in the loss of a portion of the target information and eventually tracking drift or failure. Therefore, such updating methods are doomed to drift or fail in scenes such as occlusion and deformation.

To satisfy the real-time requirement and improve tracking accuracy, we propose scene-aware adaptive updating for visual tracking via kernel correlation filters (AKCF). The AKCF uses a low complexity scale estimation method to estimate the target scale after obtaining the preliminary position of the target in the current frame by using the kernel correlation filter (KCF). Then, in the process of updating, the proposed algorithm first classifies the target scenes based on the scenes’ features. Next, for each scene, a scene-aware adaptive updating mechanism is proposed to update the target feature template and the learning rate to obtain the new kernel correlation filter. Finally, the updated kernel correlation filter is used to track the target in the next frame. The primary contributions of our proposed algorithm are summarized as follows:

1. A scene-aware adaptive updating mechanism

The conventional algorithms based on correlation filters utilize the fixed updating method. When a deformation occurs, with the fixed learning rates, the algorithms cannot rapidly learn the information of the target variation, thus cannot adapt to the drastic changes in the target appearance. When the occlusion occurs, because the holistic target feature template is updated, the information of the occluded portion is updated to the filter, resulting in tracking drift or failure. Because of this, we propose scene-aware adaptive updating for visual tracking via kernel correlation filters. First, considering the severity of the deformation as well as the area and the duration of the occlusion, the target scenes are classified into four categories: deformation, no occlusion and no deformation, partial occlusion and long-time large-area occlusion. Then, three feature parameters: similarity, holistic response and block response are combined to determine the target scene in the current frame. Finally, for the corresponding scene, we propose the scene-aware adaptive updating mechanism to update the target feature template and learning rate to update the kernel correlation filter. This approach makes full use of the features of the target scenes to determine where the target is in the scene and then adopts the corresponding updating strategies to update the filter, making the result more reliable. 

2. A low complexity scale estimation method

The existing algorithms via correlation filter use a fixed tracking bounding box, which will lose part of the target information and lead to an inaccurate target scale. Therefore, we propose a low complexity scale estimation method to determine the target scale. Five scales are selected in total, two of which is in the direction of zooming in and two in the direction of zooming out, as well as one in the original target scale. Next, these five scale candidate samples are input to the kernel correlation filter to re-respond. For each scale, the corresponding maximum response is obtained. Additionally, based on the probability of occurrence of these five scales, five corresponding weights are assigned to the scales. Finally, the target scale is determined by combining the maximum responses and the weights. This scale estimation method has low computational complexity; therefore, it can not only estimate the scale but also ensures the tracking speed.

This paper is organized as follows: Section 2 introduces the scene-aware adaptive updating for visual tracking via correlation filters, Section 3 shows the tracking results and experimental analysis of the proposed algorithm against existing algorithms, and Section 4 presents the study’s conclusions.

## 2. Scene-Aware Adaptive Updating for Visual Tracking via Correlation Filters

**Figure 1:**
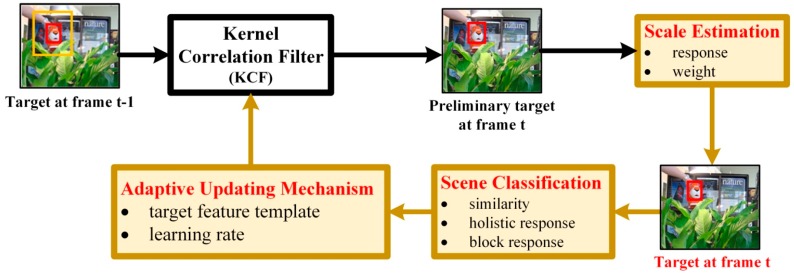
The object tracking process of our proposed algorithm.

To improve tracking accuracy under the premise of real-time tracking, we propose the scene-aware adaptive updating mechanism for visual tracking via correlation filters. The tracking process of this paper is shown in Figure 1. First, the image patch in frame *t* (labeled in the yellow block and based on the target position in the previous frame) is put to the KCF tracker trained by the target in frame *t* − 1 and the preliminary target position in frame *t* is obtained. Then, by the scale estimation, the true scale of the target is determined by combining the maximum kernel correlation filter response of the target in different scales with the corresponding assigned weight. Next, we classify the target scenes in frame *t* by three feature parameters: similarity, holistic response and block response. Then, the adaptive updating mechanism is utilized to update the target feature template and learning rate based on the determined scene. Finally, the updated kernel correlation filter is obtained to track the target in the next frame.

This section is organized as follows: in Section 2.1, we briefly introduce the construction process of the kernel correlation filter. In Section 2.2, we present the design of our low complexity scale estimation method. In Section 2.3, we describe the scene-aware adaptive updating mechanism in detail.

### 2.1. Kernel Correlation Filter

One of the greatest advantages of correlation filters is the ability to acquire abundant target and background information through dense sampling. Additionally, a correlation filter can be trained by the candidate samples obtained by dense sampling. A correlation filter can transform correlation operations in an image domain into an element-wise product operation in a Fourier domain by FFT, which greatly reduces the computational complexity and increases the tracking speed. Then, to improve the tracking performance, the CSK tracker proposed in [15] combines the kernel trick with the correlation filter. To further improve the tracking effect, the KCF tracker proposed in [25] transforms the single-channel gray feature in CSK into the multi-channel HOG [28] feature, which enhances the target description. On the basis of previous work, the basic framework of the kernel correlation filter has been developed.

Next, we briefly introduce the construction method of the kernel correlation filter (KCF) [25]. The kernel correlation filter is essentially a classifier. The classifier *f*(*x*) = *w^T^x* is trained on an image patch *x* of size *M* × *N*. The target-centered image patch is regarded as the positive sample. Through the cyclic shift of *x*, all *M* × *N* negative samples *x_m,n_* are obtained, where (*m*,*n*) ∈ {0, 1, …, *M* − 1} × {0, 1, …, *N* − 1} represents the step length of the cyclic shift. The regression labels *y*(*m*,*n*) of all samples obey the Gauss distribution, in which the target center position is set to 1 and gradually reduces to 0 with the shift to the border. Then, the classifier *f*(*x*) can be trained by minimizing the regularized risk. The minimization problem can be described by [25]:

(1) $$\underset{w}{\arg\min}\overset{}{\underset{m,n}{\sum }}{\left|\phi \left(x_{m,n}\right)\cdot w-y(m,n)\right|}_{}^{2}+\lambda ||w|{|}^{2}$$

where ϕ is the mapping to the kernel space and λ is a regularization parameter. *w* is the weight coefficient of the classifier. Here the kernel trick is introduced. The kernel function can be expressed as $\kappa \left(x,x_{m,n}\right)=\langle \phi \left(x\right),\phi (x_{m,n})\rangle$, where 〈•〉 denotes the inner product. Then the solution of the classifier can be expressed as $w=\underset{m,n}{\sum }\alpha \left(m,n\right)\phi (x_{m,n})$. Therefore, training the classifier *f*(*x*) is mapped to calculate the coefficient *α*, which can be determined by [25]:

(2) $$\alpha =F^{-1}(\frac{F(y)}{F(k)+\lambda })$$

where *F* and *F*^−1^ represent the discrete Fourier transform and its inverse respectively, and *k* can be calculated by $k=\kappa \left(x_{m,n},x\right)$.

The KCF tracker consists of the classifier coefficient α and the learned target feature template. Then in the tracking process, the target position can be obtained by calculating the classifier response in a new frame. The classifier response of the candidate *z* with the size *M* × *N* in a new frame can be calculated by [25]:

(3) $$\hat{y}=F^{-1}(F({\bar{k}}^{z})⊙F(\alpha ))$$

where the element of ${\bar{k}}^{z}\;$is ${\bar{k}}_{i}=\kappa \left(z,P^{i}\bar{x}\right)$ and *P* is the permutation matrix that cyclically shifts $\bar{x}$ by one element, $\bar{x}$ is the learned target feature template. ⊙ represents the element-wise product. Therefore, the target position of the new frame is at the location obtaining the maximum response value of $\hat{y}$.

The kernel correlation filter (KCF) can track well the position of targets in some simple scenes. However, in the tracking process, since the size of the search window at each frame is fixed, the scale of the target is not accurate. Although there are some methods to address the scale variation, the majority of them exploit the exhaustive scale estimation. Thus, these methods have high computational complexity, which will sacrifice some tracking speed. Therefore, to estimate the target scale under the premise of ensuring the tracking speed, we design a low complexity scale estimation method to solve this problem in Section 2.2.

After obtaining the target in the current frame, to adapt to the variation of the target appearance and scene in the subsequent frames, the kernel correlation filter must learn and be updated constantly. The KCF [25] algorithm always updates the holistic target feature template and the kernel correlation filter coefficient with a fixed learning rate based on the tracking results of the previous and current frame, as follows:

(4) $${\hat{x}}_{t}=\left(1-\eta \right){\hat{x}}_{t-1}+\eta \hat{x}$$

(5) $${\hat{\alpha }}_{t}=\left(1-\eta \right){\hat{\alpha }}_{t-1}+\eta \hat{\alpha }$$

where $\hat{x}$ is the target feature template of the tracking result in the current frame. $\hat{\alpha }$ is the coefficient of the kernel correlation filter trained by the tracking result in the current frame. ${\hat{x}}_{t-1}$, ${\hat{\alpha }}_{t-1}$ and ${\hat{x}}_{t}$, ${\hat{\alpha }}_{t}$ are the previous and updated target feature templates and kernel correlation filter coefficients, respectively, and *η* is the learning rate.

In the KCF [25] algorithm, the holistic target feature template is entirely updated in any scene. When the target has occlusion, the KCF will update the information of the occluded portion in the target feature template, which will lead to tracking drift or failure in subsequent frames. Additionally, *η* is always a fixed value, which indicates the target feature template and the kernel correlation filter coefficient are updated at the same rate. However, if the target has deformation, the learning rate must be higher to rapidly learn the variation information of the target. If the target has occlusion, the learning rate must be lower to avoid learning the occluded portion. Therefore, a fixed learning rate cannot adapt to the tracking process requirement of different scenes. To solve these problems, we propose the scene-aware adaptive updating mechanism in Section 2.3.

### 2.2. Low Complexity Scale Estimation Method

**Figure 2:**
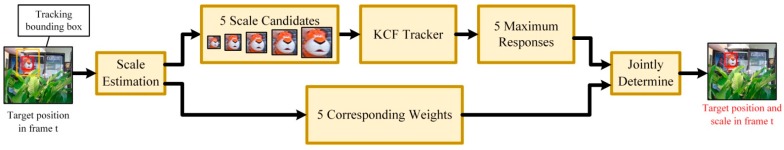
The process of the proposed scale estimation method.

In the target tracking process, the target may have scale variation. The traditional tracking algorithms based on correlation filters have a fixed-size tracking bounding box. When scale variation occurs, the fixed-size tracking bounding box will lose the true target information, resulting in tracking drift or failure. Thus, these algorithms cannot adapt to the scale variation. However, because of the uncertainty of the target scale in the tracking process, if the exhaustive scale estimation method is adopted, it will greatly increase the computational complexity and affect the tracking speed. Additionally, the probability of occurrence of the different scale variations is different. Therefore, we propose a low complexity method for scale estimation. The scale estimation process is shown in Figure 2. To estimate the true scale of the target, the maximum kernel correlation filter (KCF) response *r_i_* of the target in *i*-th scale is multiplied with the corresponding weight *δ_i_*, as shown:

(6) $$R_{i}=r_{i}\times \delta_{i}\;i=\{1,2,3,4,5\}$$

where *R_i_* represents the final response of *i*-th scale. Finally, the scale with the maximum *R_i_* will be determined to be the true scale in the current frame.

First, we assume that the initial target size is *s*_0_ = (*ω*,*h*). In the process of scale variation, the target may become larger (positive), smaller (negative), or remain the same. To reduce the computation complexity caused by a large number of scale estimations, two scales are selected in the direction of zooming in and two scales in the direction of zooming out, as well as one scale in the original target scale. However, when the target is in the scene with long-time large-area occlusion, the scale estimation does not make sense for tracking accuracy and consumes a lot of computational cost and affects the tracking speed. Therefore, the scale of the target is only estimated in the scenes that do not contain long-time large-area occlusion. In the process of scale estimation, a total of five scales are selected, which can be expressed as *s* = {*s*_−2_, *s*_−1_, *s*_0_, *s*_1_, *s*_2_}, where *s*_−2_ < *s*_−1_ < *s*_0_ < *s*_1_ < *s*_2_. Then based on prior research, we assume that the scale of the target between adjacent frames will not change significantly and the probability of the target maintaining the original scale *s*_0_ will be larger. Thus, the original scale *s*_0_ is given to the highest weight *δ*_0_. Likewise, the probability of the target has larger scale variation is lower. Therefore, for *s*_1_ and *s*_−1_, we give the corresponding weight *δ*_1_ less than *δ*_0_. For *s*_2_ and *s*_−2_, we give the corresponding weigh *δ*_2_ less than *δ*_1_. The weights can be expressed as *δ* = {*δ*_−2_, *δ*_−1_, *δ*_0_, *δ*_1_, *δ*_2_}, which corresponds to *s* = {*s*_−2_, *s*_−1_, *s*_0_, *s*_1_, *s*_2_}.

Additionally, for each scale of *s*, a large number of candidate samples are obtained by dense sampling and the target feature templates are obtained by extracting the HOG feature from the candidate samples. For ease of computation, these templates are normalized to a fixed size identical to the size of the correlation filter model. Then, these feature templates are placed into their respective kernel correlation filters (KCF tracker) to re-respond, and the maximum response *r_i_* at the *i*-th scale is obtained. The specific parameter settings are provided in Section 3.

### 2.3. The Scene-Aware Adaptive Updating Mechanism

After obtaining the position and scale of the target in the current frame, the kernel correlation filter need be properly updated to track the target in the subsequent frames. For any scenes, the KCF algorithm always updates the kernel correlation filter by updating the holistic target feature template with a fixed learning rate. When deformation occurs, the fixed learning rate cannot maintain pace with the speed of the target variation, resulting in the loss of a portion of the target information. Thus, when the target is in the scene of deformation, the filter requires a higher learning rate than in other scenes to rapidly learn the variation information of the target. When occlusion occurs, the KCF algorithm updates the holistic target feature template, which results to that the information of the occluded portion is updated into the template. After the occlusion disappears, the filter will not find the true target, leading to tracking drift or failure. Thus, when the target is in the scene with occlusion, the filter only needs to update part of the target feature template that is not occluded.

**Figure 3:**
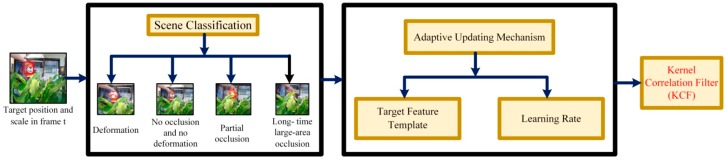
The process of the scene-aware adaptive updating mechanism.

Therefore, to make the algorithm well adapt to the target tracking in different scenes, we propose the scene-aware adaptive updating mechanism. As shown in Figure 3, first, the target scenes are classified into four categories: deformation, no occlusion and no deformation, partial occlusion and long-time large-area occlusion, and then determines in which scene the target of the current frame is located. Next, based on the proposed adaptive updating mechanism, for the determined scene, the target feature template and learning rate are updated with the corresponding strategies. Finally, the updated kernel correlation filter is obtained for the target tracking in the next frame.

#### 2.3.1. Target Scene Classification

**Figure 4:**
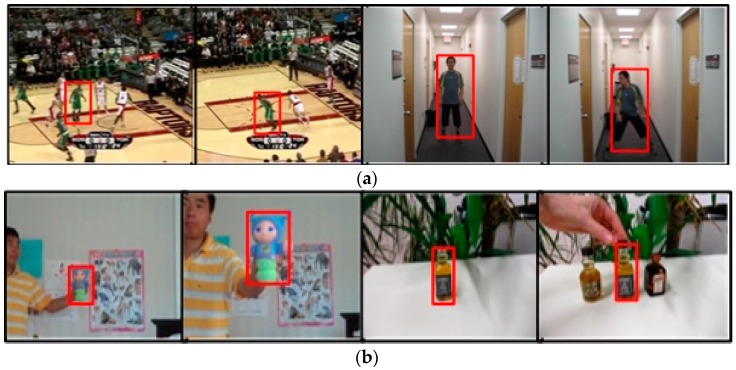

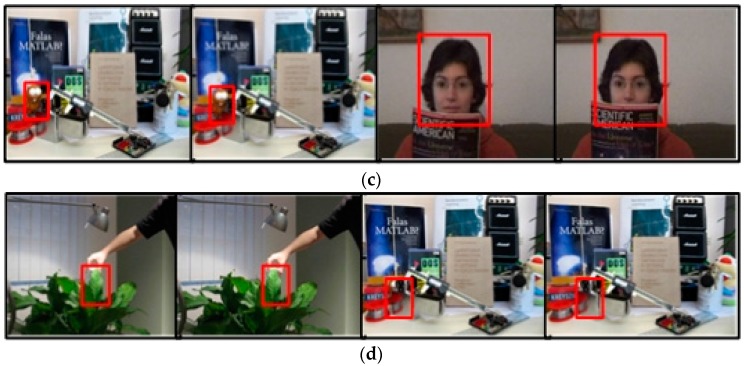
The scene classification of target (**a**) Deformation (**b**) No occlusion and no deformation (**c**) Partial occlusion (**d**) Long-time large-area occlusion.

As shown in Figure 3, in the scene-aware adaptive updating mechanism, the target scenes are first classified. Considering the severity of the deformation as well as the area and duration of the occlusion, the target scenes are divided into the following four categories: deformation, no occlusion and no deformation, partial occlusion and long-time large-area occlusion. The specific classification scenes are shown in Figure 4. Deformation refers to a drastic non-rigid deformation of target. Moreover, we assume that deformation and occlusion do not exist simultaneously in a scene during the tracking process. This is because when the target has both occlusion and deformation, it is difficult to track the target according to the information we have studied before.

#### 2.3.2. Decision Method of Target Scenes

**Figure 5:**
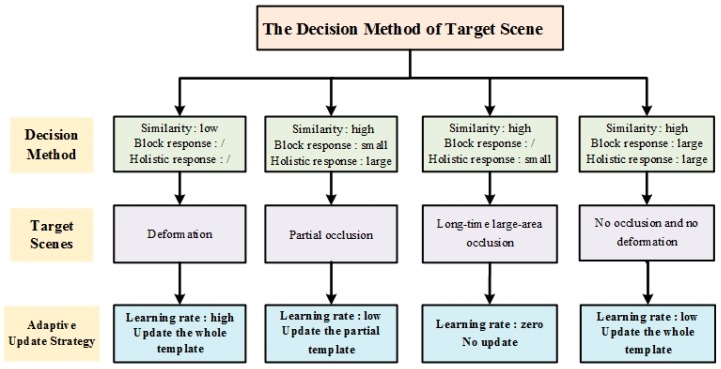
The decision method of target scene.

In this section, we describe the decision method of target scenes in the scene-aware adaptive updating mechanism in detail. As shown in Figure 5, in the entire process of deciding the scene, the three feature parameters including similarity, holistic response and block response are used. Next, we illustrate the meaning of these three parameters and the methods to obtain them.

1. Similarity

Similarity refers to the similarity degree between the target tracking results of the current frame and the previous frame. We can directly judge whether the target is in a deformation scene based on the similarity. When the similarity is low, the target has dramatic deformation and vice versa.

Similarity can be obtained by exploiting the response of the kernel correlation filter. First, a kernel correlation filter is trained individually by the target feature template of the tracking result in each frame, which can be called a similarity classifier. Then, the similarity will be obtained by putting the target feature template in the current frame into the similarity classifier.

2. Block response

**Figure 6:**
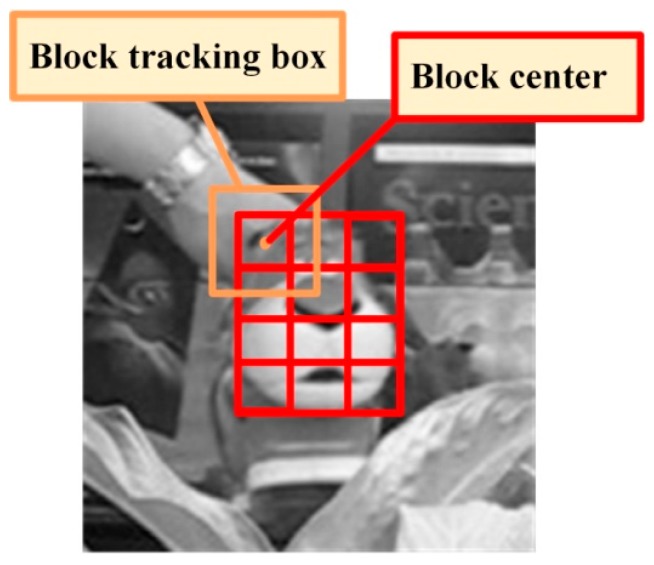
The method of block division.

When occlusion occurs, the partial occlusion always appears first. To obtain the local information of the target, the target is divided into blocks. As shown in Figure 6, for each block, the block kernel correlation filter is trained by the target feature templates obtained by dense sampling in the block tracking box. Thus, the block response can be obtained by putting the target feature template of each block in the current frame into the block kernel correlation filter. Then, we can exploit the block response to determine the partial occlusion. When the block response is higher, the block is less likely to be occluded. However, when the block response is low, it is likely that the block is being occluded.

The specific block division method is shown in Figure 6. Firstly, the algorithm needs to determine whether the target should be divided according to the size of the target. When the target is too small, the block division is meaningless and will increase the computational complexity. We specify that when the width and height of the target are both greater than 15 pixels, the target is divided. In addition, we guarantee that the width and height of each block are greater than or equal to 15 pixels. Simultaneously, to control the complexity, the number of blocks is not greater than 6 × 6.

3. Holistic response

For each frame, the KCF tracker is trained by the holistic target feature template. Then the holistic response can be obtained by putting the holistic target feature template in the current frame to the KCF tracker. When the holistic response is low, the target may experience the great change of state such as large-area occlusion.

After obtaining these three feature parameters, the target scene in the current frame can be determined by combining the three parameters, as shown in Figure 5. Next, we will illustrate the specific scene decision conditions.

When the target is in a scene with deformation, the similarity is low. Through a large number of experiments, we find the threshold to determine whether the target has deformation and set it as the similarity threshold. Then, if the similarity *ς_t_* of frame *t* satisfies:

(7) $$\varsigma_{t}<\varsigma$$

it is determined that the target deformation occurs at that time. *ς* is the threshold of similarity.

When the target is in the scene with partial occlusion, the similarity is high, the holistic response is large, but the response of some block is small; thus, by combining the feature parameters, the occlusion of the blocks in frame *t* can be judged. In the updating mechanism, due to the differences in the target and block sizes in different video sequences, the magnitude between the target and block response in the KCF tracker is significantly different. It is difficult to use a fixed threshold to determine the decision condition. Thus, the adaptive thresholds are repeatedly exploited and the maximum response value of all the previous frames is regarded as a benchmark. Then, if the response value $B_{t}^{i}$ of block *i* in frame *t* satisfies:

(8) $$B_{t}^{i}<B_{t-1}\times p_{0}\cap B_{t}^{i}<p_{1}\times A_{t}$$

it is determined that the partial occlusion occurs at that time. $B_{t-1}$ is the dynamic threshold that represents the maximum value of the block response from the 1*_th_* to (*t* − 1)*_th_* frame. *p*_0_ is the proportion factor of this threshold in satisfying the partial occlusion condition. *A_t_* represents the average value of all block responses in frame *t*. *p*_1_ is the proportion factor of the response mean when the partial occlusion condition is satisfied.

When the target is in the scene with long-time large-area occlusion, the similarity is high while the holistic response is small. With the occlusion area gradually increasing, the holistic response of the target in the kernel correlation filter is gradually decrease. Then, when the holistic response value *r_t_* of the target in frame *t* satisfies:

(9) $$r_{t}<R_{t-1}\times p_{2}$$

it is determined that the target is experiencing long-time large-area occlusion. $R_{t-1}$ is the dynamic threshold that represents the maximum value of the holistic response from the 1*_th_* to (*t* − 1)*_th_* frame. *p*_2_ is the proportion factor of this threshold in satisfying the long-time large-area occlusion condition.

The scene with long-time large-area occlusion may disappear after a period of time. To adapt to this change of target scene, our algorithm must be able to timely detect the disappearance of the long-time large-area occlusion and change the updating strategies correspondingly. First, the algorithm records whether there is a large-area occlusion in the previous frame. If the large-area occlusion occurs in the previous frame and maintains in the current frame, the target scene will be determined to be long-time large-area occlusion and the updating strategy remains. If the large-area occlusion disappears in the current frame, the updating strategy must be changed. When the large-area occlusion disappears, the holistic response of the target begins to increase gradually and the similarity is also increasing. Then, the large-area occlusion has disappeared when the holistic response value *r_t_* of the target in frame *t* and the similarity *ς_t_* of frame *t* satisfy:

(10) $$r_{t}>R_{t-1}\times p_{3}\cup \varsigma_{t}<\varsigma_{T-1}\times p_{4}{}_{}$$

where *ς_T_*_−1_ is the dynamic threshold that represents the maximum value of the similarity from the 1*_th_* to (*t* − 1)th frame. *p*_3_ and *p*_4_ are the proportion factors of the corresponding thresholds. When none of the judgment conditions of the above three scenes is established, we can determine that the target of the current frame is in the scene with no occlusion and no deformation.

#### 2.3.3. Adaptive Updating Strategy

After the target scene is determined, our algorithm exploits the proposed adaptive updating mechanism to update the target feature template and obtain the learning rate to suit for the scene of the current frame. The specific updating strategies are shown in Figure 5.

When the target is in a scene with deformation, the updating with low learning rate will make the filter lose the appearance change information, resulting in tracking drift or failure. Therefore, to rapidly learn the target change information, we must update the holistic target feature template with a high learning rate.

When the target is in a scene with partial occlusion, the updating of the holistic target feature template will cause the information from the occluded portion to be updated in the kernel correlation filter. Therefore, to avoid learning the information of the occluded portion, we only must update the target feature template of the blocks that are not occluded with a low learning rate.

When the target is in a scene with long-time large-area occlusion, the target is almost completely occluded. The updating of the holistic target feature template will cause all the information of the occluded portion to be updated in the filter. Thus, the filter will turn to track the occluded portion and lose the target in subsequent frames. Therefore, to make the filter can still track the true target after the long-time large-area occlusion disappears, the updating of the target feature template is suspended and the learning rate is set to zero.

When the target is in a scene with no occlusion and no deformation, there is no dramatic change in the target appearance and no interference from outside occlusion. The kernel correlation filter will be able to detect the target. Therefore, we should reduce the learning rate so the filter can continue to detect the target based on the current state. Besides, the holistic template must be updated so that the possible changes of the target can be learned by the filter at any time.

Thus, in summary, for the scenes that may occur in target tracking, the learning rate shown in Equation (11) is exploited to update the kernel correlation filter:

(11) $$\eta_{t}=\left\{ \begin{array}{ll} 0 & \mathrm{long-time}\;\mathrm{large-area}\;\mathrm{occlusion}\\ 0.015\times (\varsigma_{t}/\varsigma_{T-1})/(r_{t}/R_{t-1}) & \mathrm{deformation}\\ 0.005\times (\varsigma_{t}/\varsigma_{T-1})/(r_{t}/R_{t-1}) & \mathrm{other}\;\mathrm{scenes} \end{array}\right.$$

The relevant thresholds selection in this section will be described and discussed in detail in Section 3.

The algorithm presented in this paper is summarized in Algorithm 1.
**Algorithm 1:** The AKCF tracker1: Input: video and the target initial position; 2: Output: the target position and scale; 3: Based on the target position in the initial frame, the feature is extracted and the target initial kernel correlation filter is obtained by Equation (2); 4: For the sequent frames 5: For the new image frame, extract the new features at the target position of the previous frame and use Equation (3) to obtain the preliminary target position of the new frame; 6: Use Equation (2) to obtain the similarity filter of the first two frames; 7: Use the Equation (6) to estimate the target scale of the current frame at the new target position; 8: When the target meets the division condition, the target is divided into blocks and the corresponding parameters are obtained; 9: Use Equations (7)–(10) to determine the scene where the target is located; 10: Update the target feature template of the current frame with the adaptive updating strategies and obtain the learning rate by Equation (11) to update the kernel correlation filter based on the scene in which the target is located; 11: end

## 3. Experiments

This algorithm is implemented in a matlab2010 environment. To evaluate the performance of our proposed AKCF algorithm, we use the videos from the CVPR2013 benchmark (OTB50) [8]. Eight typical videos are selected for further analysis. The basic information of the video sequences tested and the target scenes are shown in Table 1. 

We also compare our algorithm with eight other algorithms: ASLA Tracker [11], SCM Tracker [6], L1APG Tracker [7], Struck Tracker [34], TLD tracker [35], MIL Tracker [36], KCF Tracker [25] and DSST Tracker [24]. Our algorithm is represented by AKCF and all the compared algorithms and the text documents of the tracking results are generated by the code provided by the standard library. The basic information and the primary implementation methods of the eight compared algorithms are shown in Table 2.

### 3.1. Quantitative Evaluation

#### 3.1.1. Evaluation Metric

To evaluate the performance of our algorithm, three popular evaluation metrics including center location error, tracking precision and success rate are chosen [8]: 

(a) Center Location Error

In the accuracy test of target tracking, the center location error [8] is widely used. It is defined as the Euclidean distance between the center of the tracking result and the actual center of the target. It can be expressed by:

(12) $$CLE=\frac{1}{N}\overset{N}{\underset{i=1}{\sum }}{\left\|C_{t}{}^{i}-C_{g}{}^{i}\right\|}_{2}$$

where *N* is the total number of frames of a video, $C_{t}^{i}$ is the center location of the tracking result at the *i*-th frame, $C_{g}^{i}$ represents the actual center location of the target at the *i*-th frame, and ${\left\|•\right\|}_{2}$ represents the Euclidean distance. Obviously, the smaller the average center location error of a video sequence, the higher the accuracy of the tracking algorithm. 

(b) Tracking Precision

During the calculation of the average center location error, there exists such a situation that in a few frames, one algorithm’s tracking effect is poor and even loses the target, but it tracks well in other frames. However, after averaging the center location error in all frames, the results may show that the tracking effect of this algorithm is poor. However, this result is inconsistent with the actual tracking results. Therefore, to evaluate the tracking effect more objectively and fairly, tracking precision is used to evaluate the tracking algorithms. Tracking precision is defined in a video sequence as the ratio of the frames in which the center location error is less than a fixed threshold to the total number of frames. The specific formula is as [8]:

(13) $$\begin{array}{l} CLE=\sum_{i=1}^{N}F({\left\|C_{t}^{i}-C_{g}^{i}\right\|}_{2}<threshold)\\ F(x)=\left\{ \begin{array}{ll} 1\; & x\leq threshold\\ 0\; & x>threshold \end{array}\right. \end{array}$$

where the center location error *threshold* ∈ [0, 100] and *F*(*x*) is the indicator function.

(c) Success Rate

In addition to the center location error and precision, we then use the overlap rate to evaluate the success rate [8] of the tracking algorithms. The overlap rate is defined as the ratio of the intersection of the bounding box of the tracking result and the ground truth to the union of them. It can be calculated by [8]:

(14) $$overlap=\frac{\left|R_{g}\cap R_{t}\right|}{\left|R_{g}\cup R_{t}\right|}$$

where *R_g_* and *R_t_* respectively represent the target true region and the target region obtained by the tracking algorithm, ∩ and ∪ respectively represent the region of intersection and union, |·| represents the number of the pixels within the certain region. As can be seen, the closer the overlap is to 1, the more accurate the target area is obtained by the tracking algorithm.

To be more objective to evaluate the tracking effect of the algorithms, we use the same calculation way as the precision, which is to set the threshold. Then the success rate can be defined as the ratio of the frames of the overlap rate that is greater than the threshold value to the total number of the frames in a video sequence. It is represented by [8]:

(15) $$success(\lambda )=\frac{1}{N}\overset{N}{\underset{i=1}{\sum }}F(overlap^{i}\geq \lambda )$$

where *λ* = [0,1]. Based on the success rate of the continuous threshold, the success rate plot can be obtained. We evaluate the success rate of the algorithms by the area-under-the-curve (AUC) of each success plot.

#### 3.1.2. Parameter Settings and Discussion

(a) Parameter Settings

For each test video, only the target size and location of the initial frame are given, and other information is unknown. The regularization parameter *λ* = 10^−4^ in Equation (1). For our algorithm, in the position estimation, the size of the search window is $\varpi_{s}=2.5\times t_{s}$, and *t_s_* is the target size. For the correlation filters and features, we use the same set of parameters as the KCF tracker. In scale estimation, *S* = {0.95*s*_0_, 0.98*s*_0_, *s*_0_, 1.02*s*_0_, 1.05*s*_0_}, δ = {0.95, 0.98, 1, 0.98, 0.95}. In the scene-aware adaptive updating mechanism, in Equation (7), *ς* = 0.75. In Equation (8), *p*_0_ = 0.15 and *p*_1_ = 0.6. In Equation (9), *p*_2_ = 0.25. In Equation (10), *p*_3_ = 0.3 and *p*_4_ = 0.8. For all video sequences, we perform experiments using the same parameter settings.

(b) Discussion of Parameter Threshold

Our algorithm involves many parameter thresholds. We use a large number of experiments to select a more reliable threshold so that our algorithm can track better. Next, for each parameter in the algorithm, we will show the tracking precision and success rate plot of the proposed algorithm under the multi-group thresholds. A large amount of experimental data shows that the threshold settings above make the algorithm more effective.

(b-1) The thresholds for determining a deformation

When a deformation occurs, the similarity of the tracking results in the previous frame and the current frame is used to judge the scene. When the similarity of the adjacent frames is below a certain threshold, it is determined that the target has deformation. As shown in Equation (7), *ς_t_* < *ς*. In the experiment, we set *ς* to 0.75. Then, we select four additional threshold values to compare with the threshold we established to prove the reliability of our threshold.

**Figure 7:**
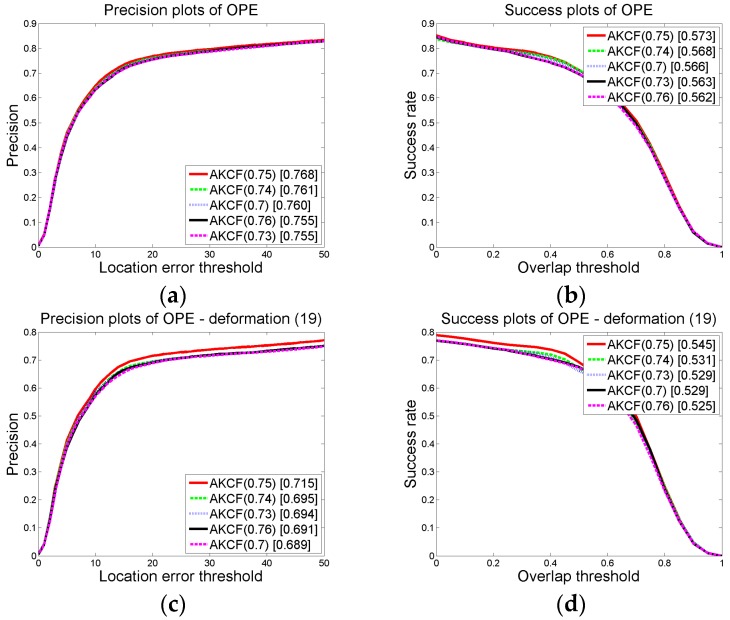
The precision (**a**) and success (**b**) plots of the proposed AKCF tracker with different threshold *ς* on 50 benchmark videos. And the precision (**c**) and success (**d**) plots of the proposed AKCF tracker with different threshold *ς* on 19 deformation videos.

When we maintain other parameter thresholds constant, we set *ς* = {0.7, 0.73, 0.74, 0.75, 0.76}. Then, the overall precision and success rate plots of the proposed algorithm on 50 videos (CVPR2013) are obtained for each threshold in *ς* and the precision and success rate plots of the 19 deformation videos are also obtained. The results are shown in Figure 7. In the graph, the numbers in brackets of AKCF (0.7), AKCF (0.73), AKCF (0.74), AKCF (0.75) and AKCF (0.76) indicate the corresponding threshold values of *ς*. The experimental results show that when the threshold *ς* varies from 0.7 to 0.75, the precision and success rate (AUC) for the 19 deformation videos show an upward trend. When the threshold *ς* is 0.76, the precision and success rate (AUC) for the 19 deformation videos begin to decrease. Therefore, when the threshold *ς* is 0.75, our algorithm is the most accurate for deformation videos and the overall precision and success rate (AUC) are also the best under this threshold.

(b-2) The thresholds for determining a partial occlusion

In judging whether the target has partial occlusion, the target that the width and height are greater than 15 pixels is divided into blocks. Then the response value of each block is used to determine whether the block is occluded. As shown in Equation (8), when the formula is satisfied, it can be determined that this block is occluded. *p*_0_ and *p*_1_ are respectively the proportion factors of the threshold *B_t_*_−1_ and *A_t_* in satisfying the partial occlusion conditions. In our algorithm, we set *p*_0_ = 0.15 and *p*_1_ = 0.6. Next, we will show the comparison results of three group threshold values. It shows that the threshold settings in Section 3.1.2-(a) can generate the best tracking results.

**Figure 8:**
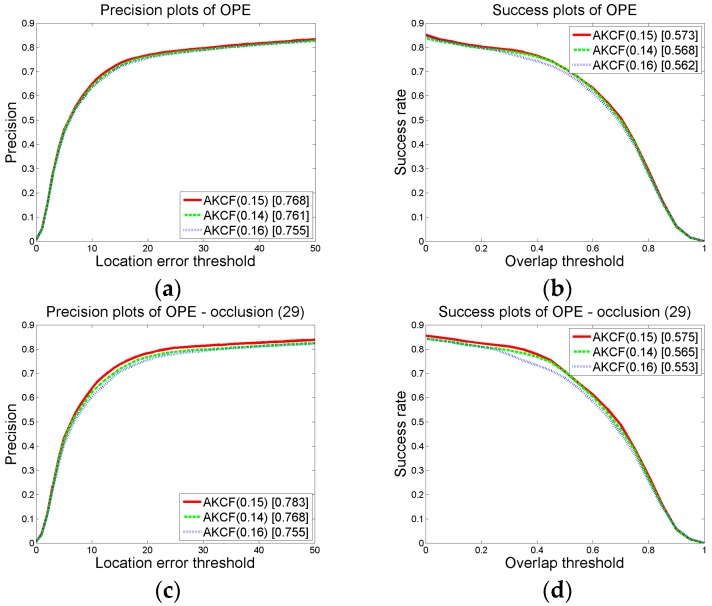
The precision (**a**) and success (**b**) plots of the proposed AKCF tracker with different threshold *p*_0_ on 50 benchmark videos and the precision (**c**) and success (**d**) plots of the proposed AKCF tracker with different threshold *p*_0_ on 29 occlusion videos.

We change the value of *p*_0_, *p*_1_ on the premise that other parameter thresholds remain unchanged. Then by the experiments, we obtain the overall precision and success rate (AUC) plots under different threshold values of our algorithm on 50 videos, as well as the precision and success rate (AUC) plots on the 29 occlusion videos. We first make sure that *p*_1_ = 0.6 remains unchanged and change the value of *p*_0_ to {0.14, 0.15, 0.16}. Then, we can obtain the results shown in Figure 8. In the graph, the numbers in brackets of AKCF (0.14), AKCF (0.15), AKCF (0.16) indicate the corresponding threshold values of *p*_0_ . We can see that when *p*_1_ keeps invariant, and *p*_0_ changes from 0.14 to 0.16, for the overall precision and success rate (AUC) on 50 videos as well as the precision and success rate of 29 occlusion videos, our algorithm achieves the optimal tracking performance when *p*_0_ = 0.15.

**Figure 9:**
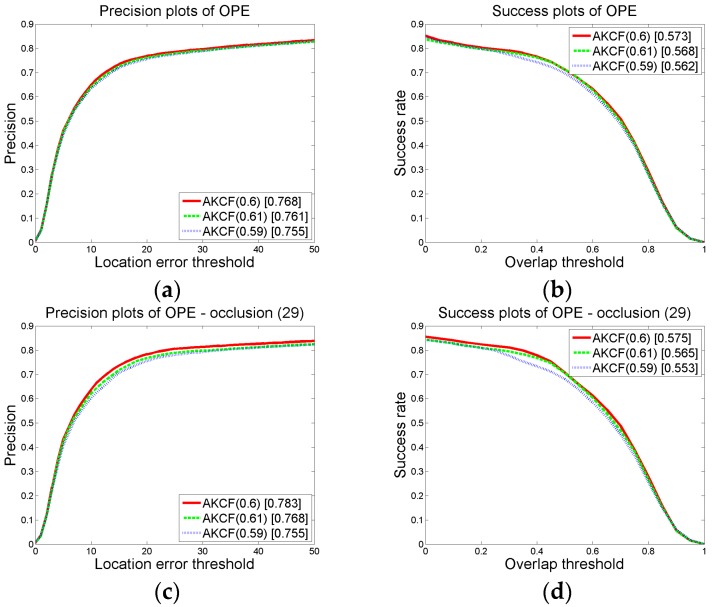
The precision (**a**) and success (**b**) plots of the proposed AKCF tracker with different threshold *p*_1_ on 50 benchmark videos and the precision (**c**) and success (**d**) plots of the proposed AKCF tracker with different threshold *p*_1_ on 29 occlusion videos.

Next, we guarantee that *p*_0_ remains unchanged and change the value of *p*_1_ to {0.59, 0.60, 0.61}. We obtain the experimental results shown in Figure 9. In the graph, the numbers in brackets of AKCF (0.59), AKCF (0.6), AKCF (0.61) indicate the corresponding threshold values of *p*_1_. We can see that when *p*_0_ remains invariant, and *p*_1_ changes from 0.59 to 0.61, for the overall precision and success rate (AUC) on 50 videos and the precision and success rate of 29 occlusion videos, our algorithm achieves superior tracking performance when *p*_1_ = 0.6.

(b-3) The thresholds for determining a long-time large-area occlusion

**Figure 10:**
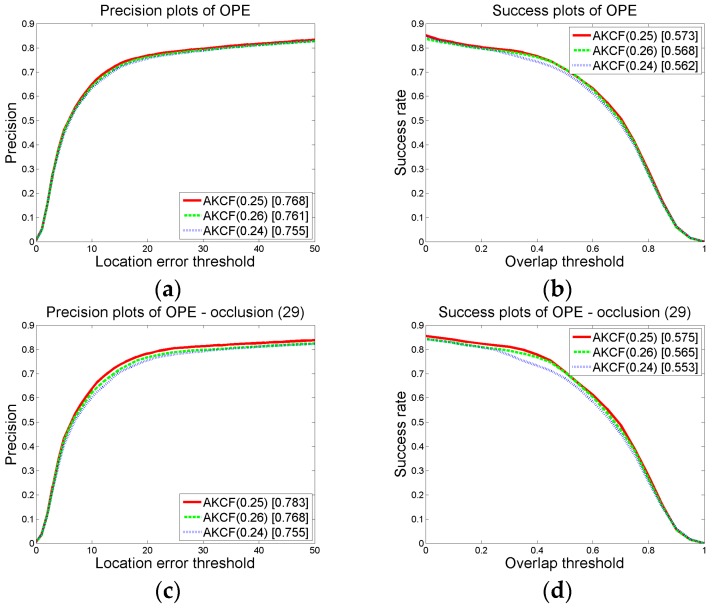
The precision (**a**) and success (**b**) plots of the proposed AKCF tracker with different threshold *p*_2_ on 50 benchmark videos and the precision (**c**) and success (**d**) plots of the proposed AKCF tracker with different threshold *p*_2_ on 29 occlusion videos.

The response of the kernel correlation filter is used to decide whether the target has long-time large-area occlusion. When the area of occlusion increases gradually, the response of the target in the filter will gradually decrease. When the response is reduced to a certain threshold, as shown in Equation (9), it can be determined that the target has long-time large-area occlusion. In Section 3.1.2-(a), we set the threshold *p*_2_ = 0.25. Next, we keep the rest of the parameter thresholds constant, and change the value of *p*_2_ to {0.24, 0.25, 0.26}. Then we perform experiments to respectively obtain the precision and success rate (AUC) of our algorithm under the three sets of thresholds. The results are shown in Figure 10. We can see that when *p*_2_ changes from 0.24 to 0.26, for the overall precision and success rate (AUC) on 50 videos and the precision and success rate (AUC) on 29 occlusion videos, our algorithm has the best tracking effect when *p*_2_ is set to 0.25. Obviously, our setting in Section 3.1.2-(a) is reliable.

(b-4) The thresholds for determining the disappearance of long-time large-area occlusion

When the target is judged to be in long-time large-area occlusion, the updating of the kernel correlation filter will be suspended. However, after the long-time large-area occlusion disappears, the algorithm must continue to learn and update the corresponding filter. Therefore, it must record that whether the target has long-time large-area occlusion in the previous frame. Then, only when Equation (10) is satisfied can we determine the long-time large-area occlusion has disappeared. In Section 3.1.2-(a), we set *p*_3_ = 0.3 and *p*_4_ = 0.8. Next, we compare the precision and success rate (AUC) of the selected three sets of thresholds to show that the threshold settings of our algorithm in Section 3.1.2-(a) can achieve superior results.

**Figure 11:**
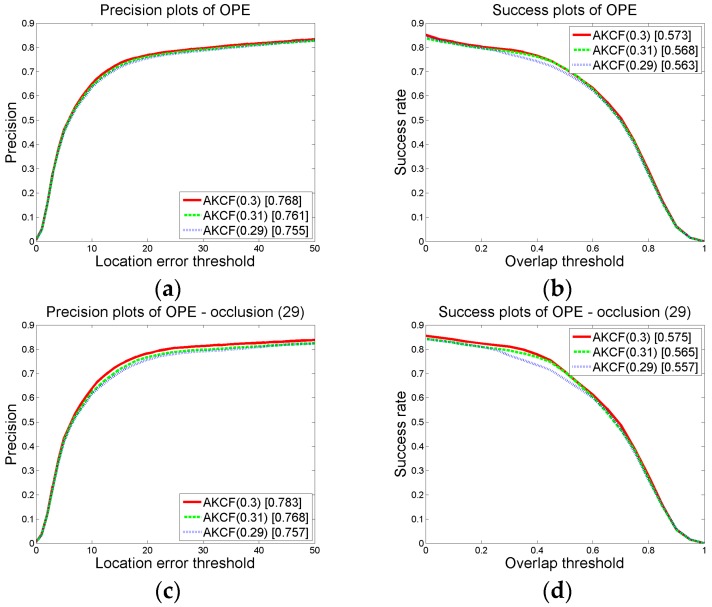
The precision (**a**) and success (**b**) plots of the proposed AKCF tracker with different threshold *p*_3_ on 50 benchmark videos and the precision (**c**) and success (**d**) plots of the proposed AKCF tracker with different threshold *p*_3_ on 29 occlusion videos.

On the premise that the other parameter thresholds remain unchanged, we keep *p*_4_ constant and change the value of *p*_3_ to {0.29, 0.30, 0.31}. We then obtain the results shown in Figure 11. The numbers in brackets of AKCF (0.29), AKCF (0.30) and AKCF (0.31) indicate the corresponding threshold values of *p*_3_. We can see that when *p*_3_ changes from 0.29 to 0.31, for the overall precision and success rate (AUC) on 50 videos and the precision and success rate (AUC) on 29 occlusion videos, our algorithm achieves the optimal tracking effect when *p*_3_ is 0.3.

**Figure 12:**
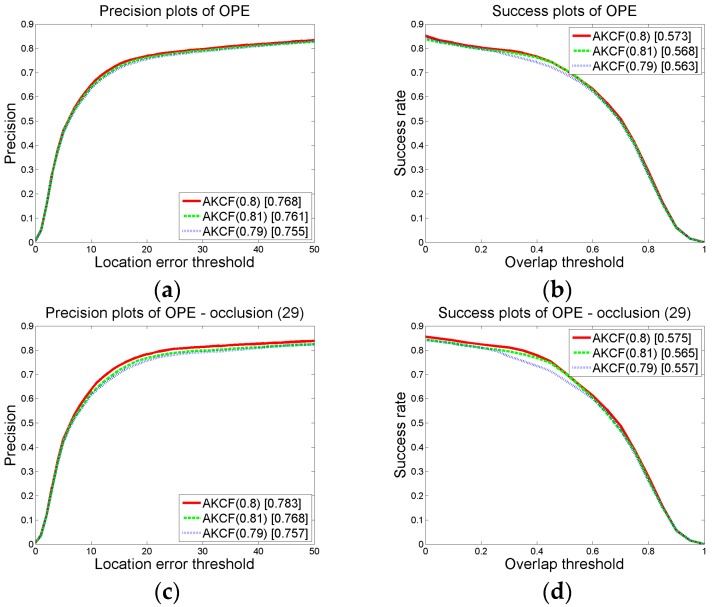
The precision (**a**) and success (**b**) plots of the proposed AKCF tracker with different threshold *p*_4_ on 50 benchmark videos and the precision (**c**) and success (**d**) plots of the proposed AKCF tracker with different threshold *p*_4_ on 29 occlusion videos.

Next, we guarantee that *p*_3_ remains unchanged and the value of *p*_4_ is changed to {0.79, 0.80, 0.81}. Through the experiments on CVPR2013, we obtain the results shown in Figure 12. The numbers in brackets of AKCF (0.79), AKCF (0.8) and AKCF (0.81) indicate the corresponding threshold values of *p*_4_. We can see that when *p*_4_ changes from 0.79 to 0.81, for the overall precision and success rate (AUC) on 50 videos and the precision and success rate (AUC) on 29 occlusion videos, our algorithm achieves the optimal tracking performance when *p*_4_ is 0.8.

#### 3.1.3. Experiment on Eight Typical Video Sequences

To analyze the performance of our algorithm compared with other eight algorithms shown in Table 2 in detail, we choose eight typical video sequences shown in Table 1 to test the tracking performance under three evaluation metrics shown in Section 3.1.1, respectively.

##### Center Location Error Comparison

**Figure 13:**
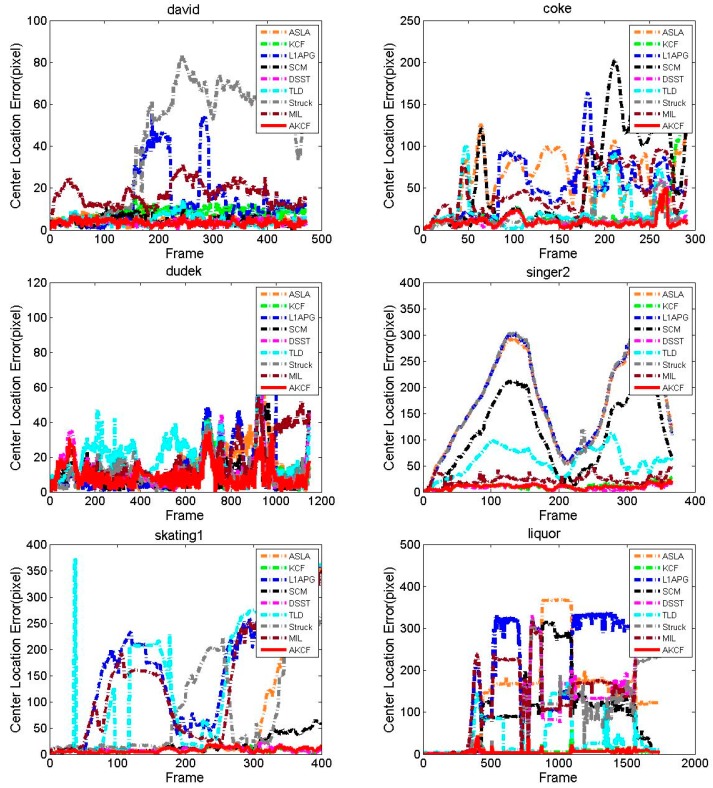

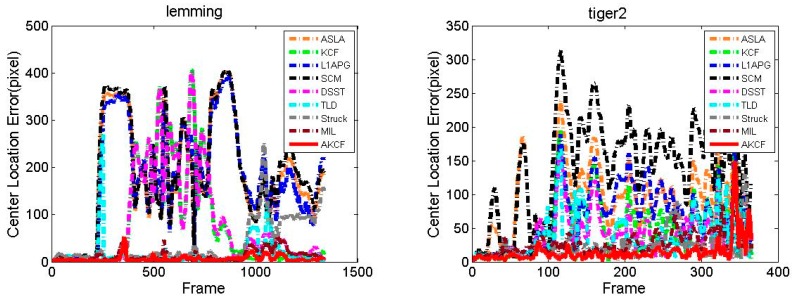
The comparison of center location error of the algorithms.

The result of the center location error of our algorithm and the other eight algorithms are shown in Figure 13. For David, the CLE of the MIL algorithm is increasing from the beginning frames and its CLE is in a very unstable state in the subsequent frames. At approximately the 150th frame, the CLE of the Struck and L1APG algorithms begins to show a sharp rise. The Struck algorithm tracking is failing in the subsequent frames. The L1APG algorithm recovers near the 230th frame, but it rises sharply near the 300th frame and it is unstable in the subsequent frames. The TLD and KCF algorithms have good performance for the first 200 frames. However, after 200 frames, when the illumination conditions in the target environment begin to change greatly, the CLE of the two algorithms is higher than our algorithm. The tracking results of the SCM and DSST algorithms are similar to our algorithm and their CLE are relatively smooth. During the entire tracking process for coke, the CLE of the MIL, TLD, SCM, L1APG, and ASLA algorithms all increases sharply multiple times, so the tracking results are very unstable. 

The DSST, Struck and our algorithm have superior tracking results and our algorithm has the lowest center location error. For Dudek, Singer2, Skating1 and Liquor, our algorithm always produces a lower CLE and a more stable tracking effect. Especially for Liquor, during the entire tracking process, all algorithms have a CLE with multiple sudden increases. However, our algorithm maintains the lowest CLE and is very stable. For Tiger2, beginning at the 20th frame, the CLE of the SCM and ASLA algorithms has continuous and very large fluctuations. Near the 100th frame, the CLE of the DSST, TLD, L1APG and KCF algorithms begins sustained and larger fluctuations. This is because near the 100th frame, the target that is out of view in the previous frames reappears, and at the time of the occlusion there are continuous occurrences of fast motion and rotation. Our algorithm maintains the lowest center location error throughout the entire process and is very stable before the 350th frame. After the 350th frame, although our algorithm appears mutations similar to the other algorithms, our algorithm can immediately revert to the lowest CLE. For Lemming, in the vicinity of the 250th frame, due to the fast motion of the target, the CLE of the TLD, L1APG, SCM and ASLA algorithms rises sharply. Then the TLD algorithm recovers while the other three algorithms fail to track the target. Near the 320th frame, because of the appearance of the long-time large-area occlusion, the DSST and KCF algorithms’ tracking begins to drift and the CLE constantly fluctuates sharply. At the 1000th frame, the CLE of the Struck algorithm rises sharply and the CLE of the MIL algorithm also increases. Only our algorithm maintains the minimum center location error and remains stable throughout the process. Table 3 shows the average center location error of the proposed algorithm and the other eight algorithms. Red indicates that the algorithm has the smallest average center location error, blue indicates that the algorithm is ranked second and green indicates the algorithm is ranked third. As can be seen for seven videos, the center location error of our algorithm is the lowest. For the average center location error of all eight videos, our algorithm is significantly lower than the other algorithms. In summary, we can see that our algorithm has higher accuracy and stability when addressing tracking in different scenes by comparing the average center location errors in Table 3.

##### Tracking Precision Comparison

**Figure 14:**
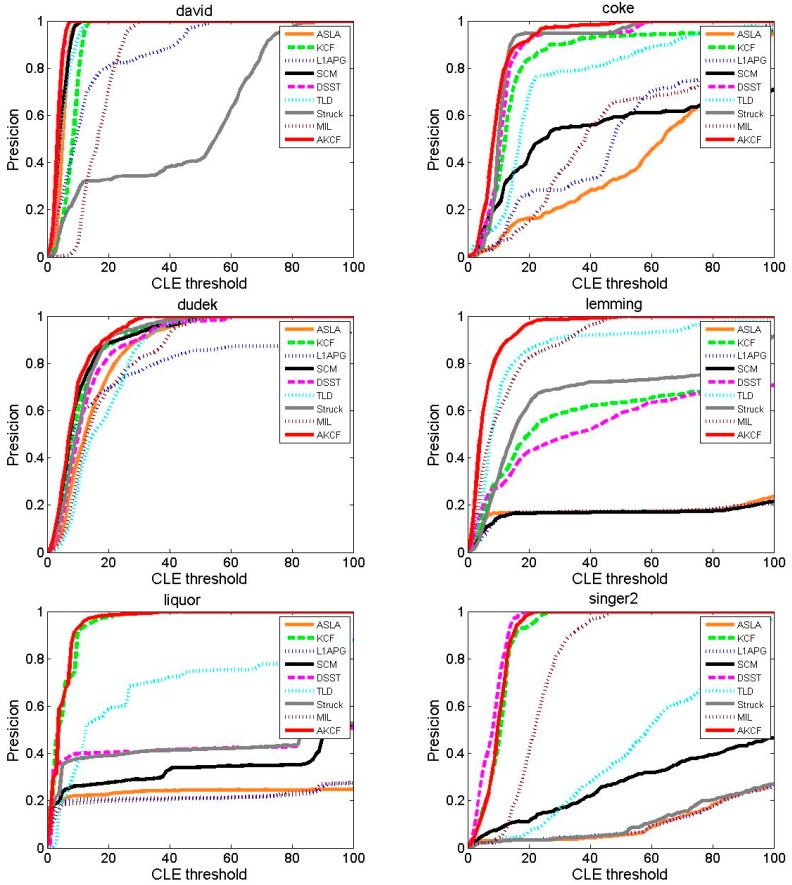

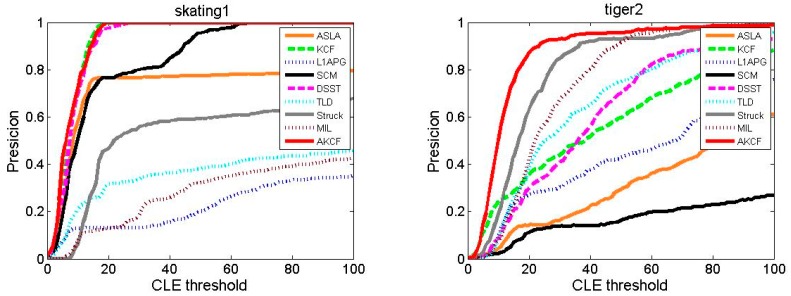
The tracking precision comparison of the eight algorithms.

The tracking precision plots of our algorithm and the other eight algorithms are shown in Figure 14. We can see that in videos except for Singer2, our algorithm first achieves 100% precision within the threshold [0, 100]. For Singer2, the tracking precision of the DSST algorithm has always been slightly higher than our algorithm in the range of [0, 20], but after the threshold of 20, the tracking precision of the DSST algorithm and our algorithm both reach 100% simultaneously. For videos David, Dudek and Lemming, our algorithm is not only the first to reach 100% precision, but it also has stable tracking precision when other algorithms are still not stable. For Coke, the tracking precision of our algorithm is lower than the Struck algorithm in the range of [17, 20], but higher than other algorithms. However, when the threshold is greater than 20, the tracking precision of our algorithm is higher than all eight algorithms. For Liquor, in the range of [0, 5], the tracking precision of our algorithm is similar to the KCF algorithm. Later, our algorithm shows slightly higher precision than the KCF algorithm, but eventually the KCF algorithm and our algorithm almost simultaneously reach 100%. For Tiger2, the tracking precision of our algorithm is always higher than other algorithms, and maintains a certain gap. 

Table 4 shows the average tracking precision of our algorithm and the other eight algorithms. The red label indicates the algorithm with the highest precision. We can see that our algorithm achieves the highest precision for seven videos. In addition, our algorithm obtains the best average tracking precision for the eight videos eight videos—significantly higher than the others. In summary, our algorithm can overcome the problem of drift in addressing video tracking with different attributes and achieve higher precision.

##### Tracking Success Rate Comparison

**Figure 15:**
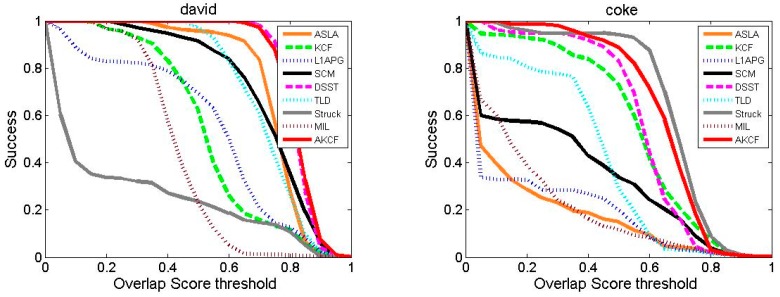

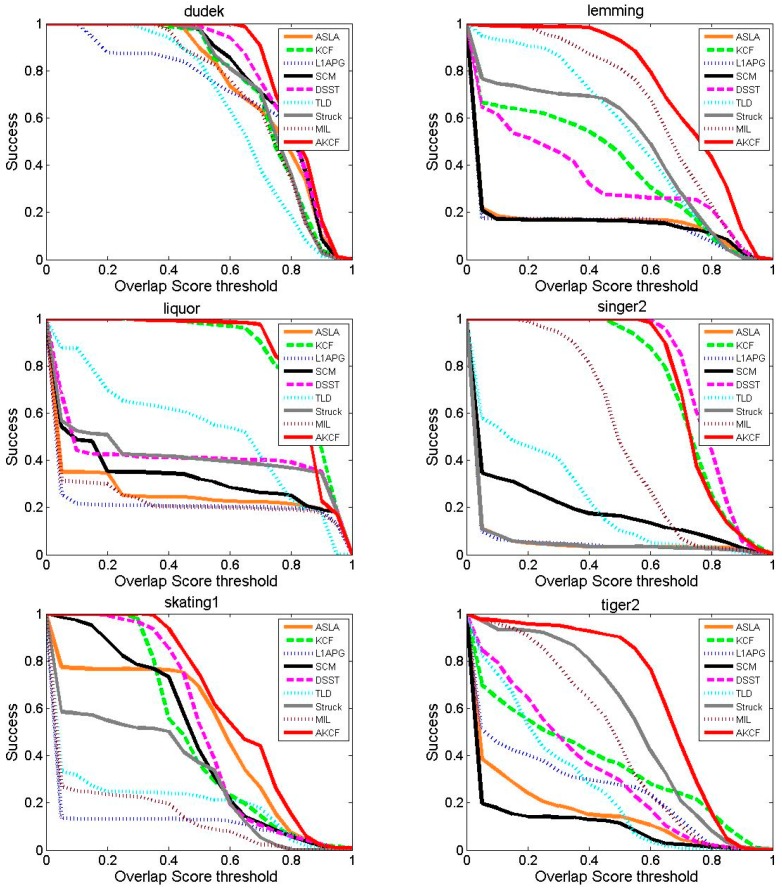
Comparison of the success rate of the algorithms.

The success rate comparison results of our algorithm with the other eight algorithms are shown in Figure 15. As can be seen, for videos Dudek, Lemming, Skating1 and Tiger2, the success rate curves and the X-axis areas of our algorithm form the largest area, that is, the highest AUC score. For video Coke, the performance of our algorithm is similar to the Struck algorithm, but the success rate of our algorithm is higher than the Struck algorithm when the threshold is in the range of [0, 0.4]. 

For David, the success rate curve of our algorithm is almost consistent with the DSST algorithm, but with the threshold [0.8, 1], our algorithm is slightly higher than the DSST algorithm. For Liquor, the success rate of our algorithm is always higher than that of all eight algorithms before 0.85, and is only slightly lower than that of the KCF algorithm in the range of [0.85, 1]. For Singer2, the success rate of our algorithm is slightly lower than the DSST algorithm, but higher than other algorithms.

Table 5 shows the average success rate of our algorithm and the other eight algorithms, in which the red label represents the highest success rate of the corresponding video. As can be seen, our algorithm shows the highest success rate for five videos, and in the other three videos, the success rate of our algorithm is only slightly lower than the best success rate. What's more, the average success rate of our algorithm for eight videos is the highest. By comprehensive comparison, our algorithm shows a higher success rate.

#### 3.1.4. Experiment on CVPR2013 Benchmark

To more objectively and comprehensively illustrate the tracking effect of our algorithm, we compare our algorithm to the other eight algorithms on CVPR2013 benchmark (OTB50). The dataset contains 50 standard test videos and 29 classic tracking algorithms.

##### Overall Performance Comparison

**Figure 16:**
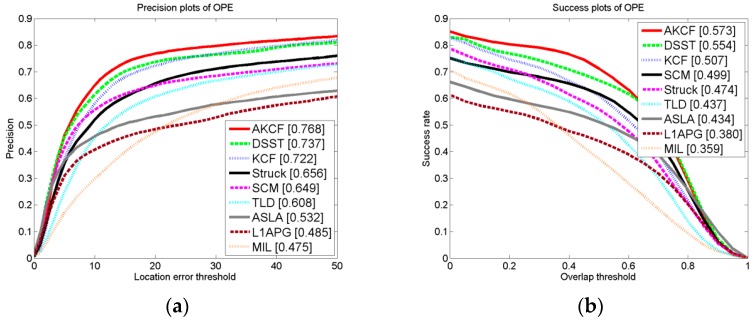
The precision plot (**a**) and success rate plot (**b**) of the algorithms on OTB50. The legend contains the area-under-the-curve score (AUC) for each tracker. The proposed AKCF algorithm performs favorably against the state-of-the-art trackers.

As can be seen from Figure 16, for the 50 test videos with 11 different attributes, the overall tracking precision and the success rate of our algorithm are the best.

##### The Comparison Results for Different Attributes

**Figure 17:**
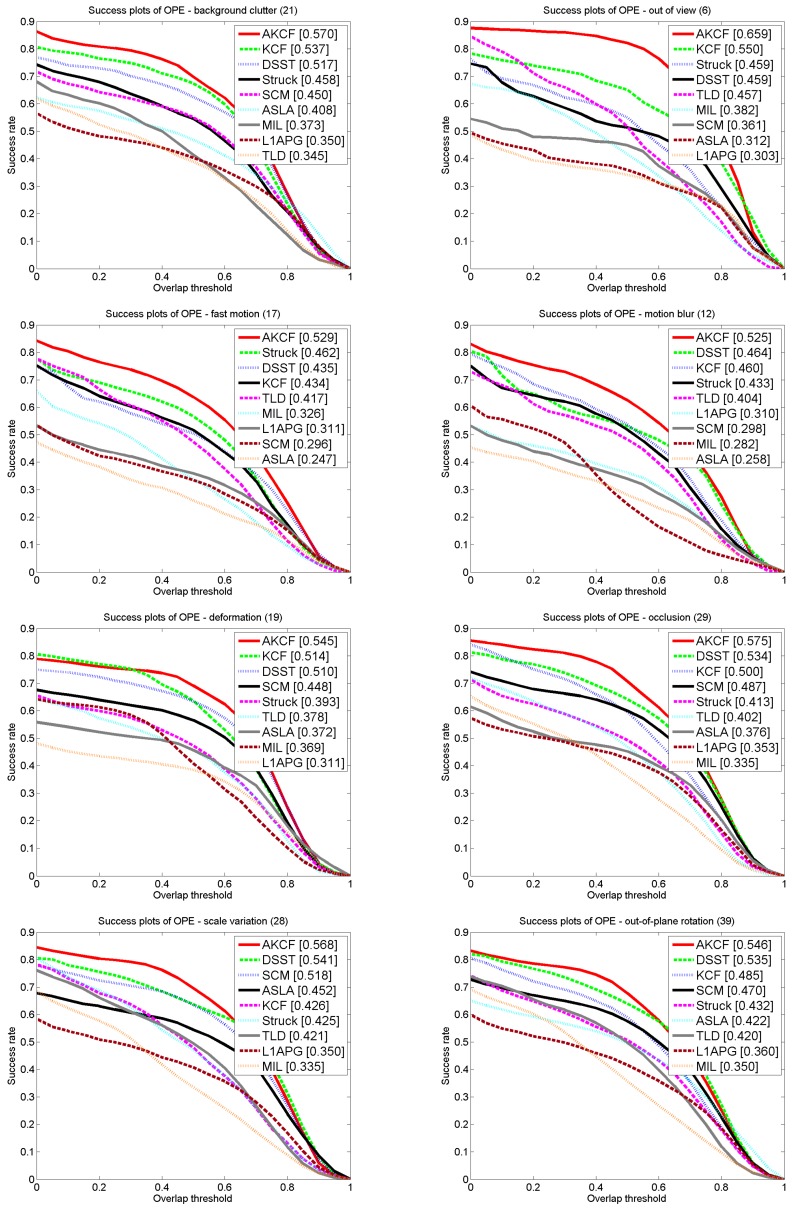
Success rate plot comparison of the algorithms for eight attributes of background clutter, out of view, fast motion, motion blur, deformation, occlusion, scale variation, and out-of-plane rotation. The legend contains the AUC score for each algorithm. The proposed AKCF tracker obtains the best performance compared with the other eight algorithms when dealing with the eight challenging factors.

After comparing the overall tracking performance of the nine algorithms on OTB50, we continue to compare the performance of the algorithms on the videos with different attributes. In the 50 videos, there are 11 different attributes that represent the problems that may be encountered during tracking, such as scale variation, occlusion, and deformation. These video attributes can assist us in better evaluating the tracking algorithms. Therefore, in Figure 17, we selected eight attributes of the videos to compare our algorithm against the eight other existing algorithms. These eight attributes include background clutter, out-of-view, fast motion, motion blur, deformation, occlusion, scale variation and out-of-plane rotation. The success rates of our algorithm are 57%, 65.9%, 52.9%, 52.5%, 54.5%, 57.5%, 70.6% and 67.5%, respectively, which are all higher than the existing algorithms’ success rates. Especially for the scale variation, although our algorithm uses a low complexity scale estimation method, its AUC is 4.6% higher than that of the DSST specific scale estimation algorithm, which also shows that our scene-aware adaptive updating mechanism can enhance the tracking effect. For the videos with occlusion, our algorithm is approximately 7.7% higher than the DSST algorithm that is ranked second. Because our algorithm uses the scene-aware adaptive updating mechanism, its performance when addressing the occlusion problem is much higher than the other algorithms. For the videos with the out-of-view attribute, our algorithm is superior to the second ranked KCF algorithm by approximately 19.8%. This is because when the target is in this scene the algorithm will determine the scene is a long-time large-area occlusion, and then suspend the updating of the target feature template and make the learning rate of the kernel correlation filter zero. Then, when the target reappears our algorithm can still track the target. For the videos with the fast motion attribute, our algorithm is superior to the second ranked Struck algorithm by 14.5%. This is because our algorithm determines the learning rate by the similarity of the tracking result in the adjacent frames. When the target is in fast motion, the similarity of the tracking results in adjacent frames decreases. Then, our algorithm increases the learning rate of the kernel correlation filter to learn the variation information of the target quickly. Thus, our algorithm can achieve more accurate target tracking.

### 3.2. Qualitative Evaluation

**Figure 18:**
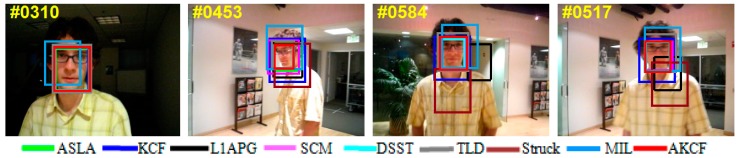
Tracking results on David.

**Figure 19:**
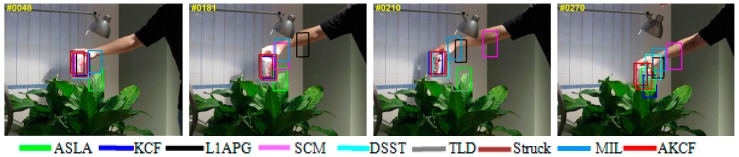
Tracking results on Coke.

**Figure 20:**
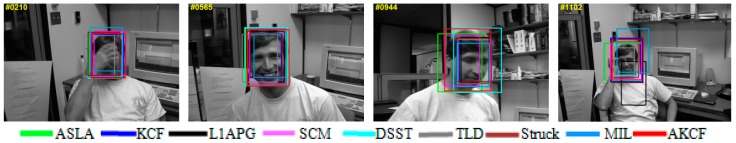
Tracking results on Dudek.

**Figure 21:**
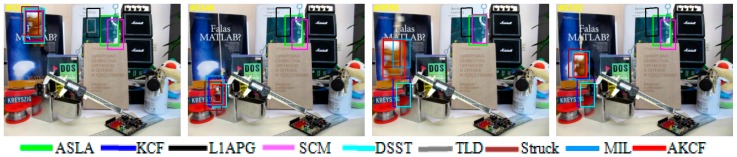
Tracking results on Lemming.

**Figure 22:**
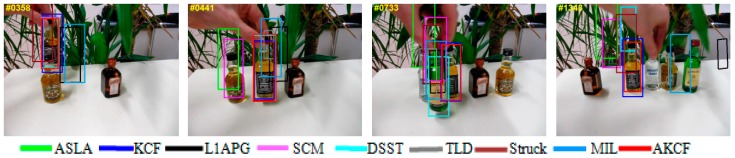
Tracking results on Liquor.

**Figure 23:**
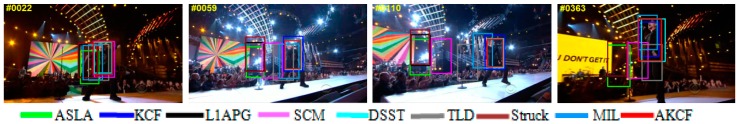
Tracking results on Singer2.

**Figure 24:**
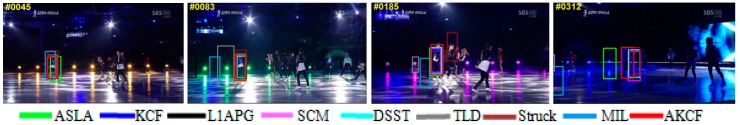
Tracking results on Skating1.

**Figure 25:**
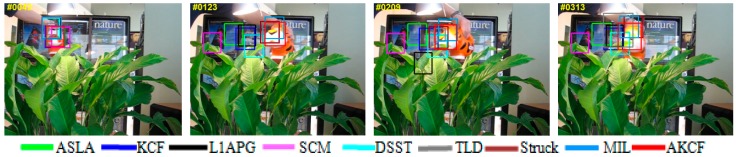
Tracking results on Tiger2.

The qualitative analysis results of our algorithm and the other eight algorithms against the eight test videos are shown in Figure 18, Figure 19, Figure 20, Figure 21, Figure 22, Figure 23, Figure 24 and Figure 25. These videos shown in Table 1 contain almost all possible problems that could be encountered. We analyze the tracking performance of the nine algorithms against the eight videos individually.

In David, the target David moves slowly from a weak light condition to a strong light condition and his pose also changes. In the 310th frame, other algorithms can track the target, but the tracking bounding box of the MIL algorithm shifts to the left slightly. The tracking results of the nine algorithms are shown in Figure 18. Subsequently, the target reaches the bright position and is experiencing out-of-plane rotation as well as scale variation. At the 453th frame, the Struck and MIL algorithms appear to drift upward and downward, respectively. The tracking bounding boxes of the KCF and L1APG algorithms cannot change adaptively with the change of the target scale. Then, the target continuously experiences an out-of-plane rotation, scale variation and changes in pose, causing the Struck and L1APG algorithms to fail and the MIL algorithm to drift. The tracking bounding box of the KCF algorithm is fixed, resulting in inaccurate tracking. Throughout the process, the tracking performance of our algorithm is similar to the DSST and SCM algorithms, which continuously track the target accurately.

In Coke, the target moves rapidly while experiencing in-plane rotation and occlusion frequently. In the 48th frame, the target reappears from the position of the occlusion by leaves in the previous frames, leading to the tracking bounding boxes of the ASLA, TLD, MIL algorithms are still remaining at the position of occlusion. These three algorithms are all tracking failure. Subsequently, the target continuously undergoes occlusion and in-plane rotation as well as fast motion. In the 181th frame, the tracking bounding boxes of the L1APG and SCM algorithms both lose the target. At the 210th frame, only our algorithm, and the Struck, DSST, KCF algorithms still track the target, and our algorithm and the Struck algorithm are more accurate. Until the 270th frame, the target reappears from the position of the occlusion, leading to the tracking bounding boxes of the DSST and KCF algorithms are separated from the target. Then, only our algorithm and the Struck algorithm continue to follow the target, and are more accurate.

For Dudek, near the 210th frame, the target is experiencing occlusion by hands and the TLD and MIL algorithms begin to drift. Then, the target is moving slowly with the variations in facial morphology and scale. At the 565th frame, the tracking bounding boxes of the Struck, MIL, and KCF algorithms cannot adaptively change with the variation of the target scale, thus these tracking is not accurate. Then, near the 944th frame, the target is experiencing in-plane rotation, leading to the DSST, ASLA, SCM algorithms all deviate from the target. The tracking bounding boxes of the Struck, KCF, MIL, L1APG and TLD algorithms are slightly smaller than the actual target scale and slightly offset to the right, so the track is not accurate. However, our algorithm is continuously tracking the target accurately. Subsequently, near the 1101th frame, due to occlusion by hands and glasses, the tracking drift of the ASLA, MIL, L1APG algorithms is very serious and the tracking bounding boxes of the Struck, KCF, and TLD algorithms are not accurate in scale. In the end, only our algorithm and the DSST algorithm track more accurately while the SCM algorithm is slightly inferior to these two algorithms.

For Lemming, we intercept the partial video frames that include scenes of fast motion, partial occlusion, and long-time large-area occlusion to analyze the tracking effect of the algorithms. The tracking results are shown in Figure 21. Before the 242th frame, the target has just undergone fast motion, and the TLD, L1APG, ASLA, and SCM algorithms fail. The Struck algorithm is offset to the left and the size of the MIL algorithms’ tracking bounding box is inaccurate. Near the 340th frame, the target experiences long-time large-area occlusion. The Struck, MIL, KCF and TLD algorithms all have different degrees of offset, but only our algorithm and the DSST algorithm continue to follow the target. In the 382th frame, the KCF and DSST algorithms fail when the target reappears. 

The Struck algorithm still has offsets, but the TLD algorithm recovers to track the target by self-detection. Then, the target continues to experience fast motion and partial occlusion. At the 550th frame, the MIL algorithm begins to drift and the size of the STRUCK and TLD algorithms’ tracking bounding boxes is inaccurate. Finally, in the entire process, only our algorithm tracks more accurately not only the target location but also the scale. In Liquor, different liquid bottles are placed on the table. First, the target moves around another bottle and undergoes partial occlusion as well as scale variation. As shown in the 358th frame, the MIL and L1APG algorithms lose the target and the Struck algorithm severely deviates from the target. Due to the fixed tracking bounding box, the KCF algorithm cannot change with the target scale variation adaptively, so the tracking is not accurate. Subsequently, the target continues to move left and part of that is in out-of-view. Then the target recovers to the initial position, as shown in the 441th frame. The ASLA and SCM algorithms lose the target. The MIL, L1APG and TLD algorithms seriously deviate from target. The tracking scales of the KCF and Struck algorithms are not accurate enough because the tracking bounding boxes cannot surround the whole target. Only our algorithm and the DSST algorithm track more accurately. Next, new liquid bottles are emerging and the shapes are similar to that of the target. Near the 733th frame, when the target is occluded by the green bottle that is in front of the target, the DSST algorithm turns to track the green bottle. Only the KCF algorithm and our algorithm continue to track the target. In the subsequent tracking process, the target constantly undergoes occlusion and in-plane rotation. However, until the end of the video, as shown in the 1343th frame, only our algorithm and the KCF algorithm are continuously tracking the target. Our algorithm is more accurate for the target scale. In summary, our algorithm shows higher accuracy in the entire process.

In Singer2, the target is undergoing severe deformation and the illumination conditions are constantly changing. At the 22th frame, the Struck, ASLA, MIL and L1APG algorithms begin to drift. Subsequently, the target continues to have deformation substantially. As shown in the 59th frame, the SCM algorithm fails and the TLD algorithm occurs severe deviation, while the size of the KCF algorithm’s tracking bounding box is not accurate enough. Next, constant variation of the illumination conditions, severe deformation of the target and rotation of the camera lens cause the TLD algorithm to fail, as shown in the 110th frame. Finally, until the last frame of the video, the Struck, L1APG, ASLA, SCM and TLD algorithms fail. The MIL algorithm is offset to the upward. The size of the KCF algorithm’s tracking bounding box is not accurate enough. Ultimately, only our algorithm and the DSST algorithm track more accurately.

In Skating1, the target is constantly undergoing severe deformation and the illumination conditions are changing constantly. Near the 45th frame, the target is turning around, that is, the target is experiencing the out-of-plane rotation. As can be seen, the ASLA algorithm occurs deviation, and the tracking bounding boxes of the MIL and Struck algorithms are not accurate enough. Subsequently, after the target undergoes the successive 360 degrees of rotations, as shown in the 83th frame, the L1APG, MIL and TLD algorithms fail. Subsequently, in the rotation process, the target is occluded by another contour-similar companion, as shown in the 185th frame. Then, when the occluding person leaves, the Struck algorithm turns to track the occluding person and leaves the target. Other algorithms can track the target, but our algorithm is more accurate both in location and scale. Until the final part of the video, the target is continuously rotating violently, and the illumination conditions in the environment are changing, as shown in the 312th frame. The TLD, L1APG, MIL and ASLA algorithms all completely lose the target. The Struck, DSST, and SCM algorithms are almost completely out of the target. Only our algorithm and the KCF algorithm continue to follow the target, and our algorithm has the more accurate location and scale.

In Tiger2, the target moves from left to right and then returns. At the 42th frame, the Struck, MIL, DSST, TLD, L1APG and SCM algorithms all begin to drift, and only our algorithm and the KCF and ASLA algorithms track the target accurately. Subsequently, the target is constantly being occluded by leaves as well as being affected by deformation and rotation. At the 123th frame, the SCM, ASLA, L1APG, KCF and DSST algorithms all deviate from the target and the tracking fails. The Struck, TLD and MIL algorithms sustain a certain degree of drift; only our algorithm tracks the target accurately. The target continues to have in-plane and out-of-plane rotation and occlusion by leaves. At the 209th frame, the SCM, ASLA, L1APG, KCF, DSST and TLD algorithms all fail. The Struck and MIL algorithms are offset from the target. Only our algorithm tracks the target exactly. The target moves in the same manner until the 313th frame. While the other algorithms experience tracking drift or even failure, our algorithm tracks the target accurately.

### 3.3. Comparison of Tracking Speed

After analyzing the tracking accuracy of different algorithms, we compare and analyze the tracking speed of our algorithm and the several existing algorithms. We run the algorithms on the 50 sequences and calculate the average running speed. The experiments runs on a PC with 3.20 GHz CPU, 4.0 GB RAM and Windows 10 operating system. The results of the average tracking speed are shown in Table 6. As can be seen, the average tracking speed of our algorithm is 26.17 fps for all 50 videos. For the other seven algorithms in Table 6, the CSK [15] algorithm obtains the fastest speed, and the tracking speeds of the KCF [25] and TLD [34] algorithms are both higher than our algorithm. However, these algorithms do not estimate the target scale, and the scale estimation will greatly reduce the tracking speed of the algorithm. Compared with the algorithm L1APG [7], SCM [6] and ASLA [11] that have the scale estimation, the tracking speed of our algorithm is greatly improved, and the real-time tracking of the target can be achieved.

### 3.4. Discussion

In this section, we discuss the tracking results for our algorithm and the other eight algorithms in detail.

#### 3.4.1. Scale Variation

Scale variation is a frequently occurring problem during target tracking. If the tracking algorithm cannot address the problem of scale variation promptly during the tracking process, the tracking is likely to eventually drift or fail. In the tracking algorithm, the computational load of scale estimation is very large, resulting in low tracking speed. Therefore, to reduce computational complexity and guarantee tracking speed, a low complexity scale estimation method is adopted in this paper. Five scales are used to estimate the target size. However, the target size can be accurately estimated, and the tracking speed is guaranteed. In Dudek, with the change of the target scale, the tracking bounding boxes of the Struck, KCF, MIL, L1APG and TLD algorithms cannot accurately and adaptively change, but our method of scale estimation is always accurate. In Liquor, the target changes in scale when it moves. We can see that the tracking bounding box’s scale of the KCF and Struck algorithms are inaccurate; our algorithm has superiorly accurate scale estimates during the entire process. Since DSST is a tracking algorithm designed to address scale variation, it is accurate for target scale estimation. However, when the DSST algorithm encounters more complex problems such as occlusion or long-time large-area occlusion, it will fail quickly. Overall, our algorithm can adapt to the scale variation of the target during the entire process and has more stable and accurate tracking effects.

#### 3.4.2. Partial or Long-Time Large-Area Occlusion

In the process of visual tracking, occlusion may easily occur, including not only partial occlusion but also long-time large-area occlusion. If the tracking algorithm does not address the occlusion immediately and effectively in the tracking process, it will drift or fail. In this paper, the scene-aware adaptive updating mechanism we proposed can effectively solve the various occlusion problems. In Dudek, the target experiences partial occlusion by a hand and glasses. The ASLA, MIL and L1APG algorithms experience severe drift. In Liquor, when the target experiences partial occlusion by another bottle, the MIL, L1APG and Struck algorithms experience tracking drift or failure. In coke and lemming, there is long-time large-area occlusion. In Coke, the target is completely occluded by leaves. Then, when the target reappears, the MIL, ASLA, and TLD algorithms stop at the shelter and fail to track. Subsequently, the target continues to experience long-time large-area occlusion by the leaves, which leads the SCM, L1APG, DSST, and KCF algorithms to fail. Ultimately, only our algorithm and the Struck algorithm continue to track the target accurately. In Lemming, the target begins to experience long-time large-area occlusion beginning at approximately the 333th frame. The Struck, MIL, KCF, and TLD algorithms all have different degrees of deviation, except that the L1APG, ASLA and SCM algorithms have failed in previous frames. Only our algorithm and the DSST algorithm continue to follow the target. After the target appears in the 382th frame, the DSST and KCF algorithms both remain at the shelter and the tracking fails. Therefore, in the entire tracking process, our algorithm has higher accuracy and robustness in solving long-time large-area occlusion.

#### 3.4.3. Out of View

It is very difficult for many algorithms to track the target that is out-of-view during the tracking process. Especially, when the target reappears, many algorithms will occur the tracking drift or even failure. But our algorithm is much better than other algorithms in addressing the videos that have the scene with out-of-view. By the quantitative analysis in Section 3.1, we can obtain that our algorithm’s success rate (AUC) achieves 0.659 in all out-of-view videos, and is higher than the second ranked KCF algorithm about 19.8%. This is mainly because when the target leaves the field of view, the algorithm will regard this scene as long-time large-area occlusion. Then, the updating of the kernel correlation filter is paused. Because the filter is not updated at this time, our algorithm can continue to track the target after the target reappears into the field of view. The scene with out-of-view happens in both Lemming and Tiger2, and we can see from Figure 18 and Figure 25 that our algorithm is more stable and accurate in tracking the target leaving the field of view.

#### 3.4.4. Fast Motion

For targets with fast motion, existing algorithms can easily fail. However, the scene-aware adaptive updating mechanism proposed by our algorithm determines the learning rate of the filter by the similarity between the tracking results in adjacent frames. When the target is in fast motion, the similarity between the two adjacent frames will decrease and our algorithm will increase the learning rate, so that the algorithm can learn the variation information rapidly and the target can be tracked more accurately. In Lemming, near the 242th frame, the target experiences fast motion. The TLD, L1APG, ASLA, and SCM algorithm all fail to track. The Struck algorithm is offset to the left and the tracking bounding box size of the MIL algorithm is inaccurate. Our algorithm and the DSST and KCF algorithms continue to follow the target. In Tiger2, the target experiences fast motion from left to right. The Struck, MIL, DSST, TLD, L1APG and SCM algorithms all experience tracking drift. Only our algorithm and the KCF and ASLA algorithms track the target accurately. The success rate (AUC) of all fast motion videos in Section 3.2 shows that our algorithm is more accurate and robust in addressing scenes with fast motion.

#### 3.4.5. Deformation and Rotation

In the tracking process, deformation often occurs simultaneously with rotation. In this paper, the similarity between the adjacent frames is used to judge this target scene. If the target has deformation and rotation, the similarity between the adjacent frames will reduce. At this point, our scene-aware adaptive updating mechanism will increase the learning rate to speed up the updating of the kernel correlation filter, so it can accurately track the target. In Singer2 and Skating1, there is severe deformation and rotation. We can see that the proposed algorithm in this paper shows satisfactory tracking results.

## 4. Conclusions

In this paper, we propose the low complexity scale estimation method and the scene-aware adaptive updating mechanism for visual tracking via correlation filters. The target scale is determined by combining the maximum kernel correlation filter response and the corresponding weight in different scale. In updating process, the target scenes are classified first and then the scene of the current frame is determined by three feature parameters from scenes’ features. Then, based on the target scene, the target feature template and the learning rate are respectively updated to obtain the new kernel correlation filter by the adaptive updating mechanism. Finally, the updated kernel correlation filter is utilized to track the target in the subsequent frames. According to the qualitative and quantitative experimental results, the proposed algorithm AKCF obtains good performance against the state-of-the-art tracking algorithms. Especially in addressing scale variation, partial or long-time large-area occlusion, deformation, fast motion and out-of-view scenes, the proposed tracker achieved an improvement of 33.3%, 15%, 6%, 21.9% and 19.8% compared to the KCF tracker on several challenging video sequences (OTB50).

## Figures and Tables

**Table 1:** Basic information of the eight test videos.

Video	Image Size	Target Size	Main Confronted Scenes
David	240 × 20	64 × 78	SV,OCC,DEF,OPR
Coke	640 × 480	48 × 80	IV,OCC,FM,IPR
Dudek	480 × 720	132 × 176	SV,IPR,OPR,FM
Lemming	640 × 480	70 × 122	SV,OCC,FM,OOV
Liquor	480 × 640	73 × 210	OPR,SV,OCC,BC
Singer2	352 × 624	67 × 122	IV,OPR,DEF,IPR
Skating	640 × 360	34 × 84	IV,SV,OCC,DEF
Tiger2	480 × 640	68 × 78	OPR,OCC,FM,IPR

**Table 2:** Basic information of the eight compared algorithms.

Algorithm	Scale Estimation	The Model Update Method
ASLA	Y	Incremental subspace learning and sparse representation combined template updating strategies
SCM	Y	The tracking results and the original template overall considered template updating strategies
L1APG	Y	The updating strategies of exploiting the similarity of the tracking results and template
Struck	N	Updating the prediction function using the update function (sample,0)
TLD	N	Exploiting the training samples generated by the evaluation results to update the object model
MIL	N	Exploiting the tracking results to update the object model online
KCF	N	Updating the object model with a fixed learning rate
DSST	Y	Updating the object model with a fixed learning rate

Y, N present scale estimation and no scale estimation, respectively.

**Table 3:** The comparison of average center location error of the algorithms.

Video	ASLA	KCF	L1APG	SCM	DSST	TLD	Struck	MIL	AKCF
David	5.07	8.06	13.95	4.34	3.65	5.12	42.80	16.86	3.49
Coke	60.17	18.65	50.45	56.81	12.79	25.08	12.08	46.72	9.90
Dudek	15.26	11.38	23.46	10.77	13.46	18.05	11.45	17.70	9.03
Lemming	178.82	77.80	177.59	185.72	81.91	15.99	37.75	12.06	6.13
Liquor	146.74	5.34	212.87	99.23	98.70	37.58	90.99	141.88	4.94
Singer2	175.28	10.33	180.87	113.63	7.77	58.32	174.32	22.53	9.69
Skating1	59.86	7.67	158.70	16.38	8.33	145.45	82.94	139.38	7.39
Tiger2	85.83	47.44	65.16	141.17	41.44	37.10	21.64	27.17	14.27
Average	90.88	23.33	110.38	78.51	33.51	42.84	59.25	53.04	8.11

Red is the best, blue is the second, green is the third.

**Table 4:** Tracking precision comparison of the algorithms.

Video	ASLA	KCF	L1APG	SCM	DSST	TLD	Struck	MIL	AKCF
David	0.944	0.915	0.857	0.952	0.959	0.944	0.571	0.828	0.961
Coke	0.404	0.818	0.510	0.528	0.868	0.746	0.875	0.532	0.897
Dudek	0.844	0.882	0.764	0.888	0.862	0.816	0.881	0.820	0.905
Lemming	0.171	0.571	0.167	0.164	0.532	0.853	0.654	0.876	0.934
Liquor	0.235	0.941	0.215	0.331	0.420	0.671	0.417	0.212	0.946
Singer2	0.097	0.892	0.097	0.263	0.918	0.420	0.106	0.772	0.899
Skating1	0.717	0.919	0.217	0.833	0.913	0.351	0.512	0.271	0.922
Tiger2	0.295	0.565	0.418	0.159	0.602	0.639	0.783	0.727	0.857
Average	0.463	0.813	0.406	0.515	0.759	0.680	0.600	0.630	0.915

Red is the best, blue is the second, green is the third.

**Table 5:** Average success rate comparison of the algorithms.

Video	ASLA	KCF	L1APG	SCM	DSST	TLD	Struck	MIL	AKCF
David	0.736	0.538	0.536	0.712	0.804	0.707	0.259	0.432	0.806
Coke	0.192	0.550	0.203	0.342	0.570	0.404	0.665	0.224	0.637
Dudek	0.725	0.720	0.682	0.756	0.774	0.640	0.723	0.698	0.798
Lemming	0.180	0.398	0.172	0.176	0.344	0.530	0.485	0.642	0.722
Liquor	0.276	0.836	0.231	0.340	0.418	0.520	0.420	0.248	0.834
Singer2	0.086	0.721	0.084	0.200	0.769	0.239	0.084	0.512	0.734
Skating1	0.501	0.491	0.143	0.471	0.526	0.221	0.328	0.161	0.624
Tiger2	0.176	0.368	0.266	0.128	0.337	0.276	0.544	0.461	0.650
Average	0.359	0.578	0.290	0.391	0.568	0.442	0.439	0.422	0.725

Red is the best, blue is the second, green is the third.

**Table 6:** The tracking speed comparison of the algorithms.

	KCF	CSK	TLD	Struck	L1APG	SCM	ASLA	AKCF
Scale estimation	×	×	×	×	√	√	√	√
Tracking speed (FPS)	157.1	221.7	28.1	20.0	2.0	0.51	8.5	26.17
